# Isolation and Characterization of High-Temperature-Tolerant Mutants of *Bradyrhizobium diazoefficiens* USDA110 by Carbon-Ion Beam Irradiation

**DOI:** 10.3390/microorganisms12091819

**Published:** 2024-09-02

**Authors:** Katsuya Satoh, Kiyoko Takeda, Ikuko Nagafune, Wan Dalila Wan Chik, Naoko Ohkama-Otsu, Shin Okazaki, Tadashi Yokoyama, Yoshihiro Hase

**Affiliations:** 1 Takasaki Institute for Advanced Quantum Science, National Institutes for Quantum Science and Technology (QST), 1233 Watanuki-machi, Takasaki 370-1292, Gunma, Japan; sato.katsuya@qst.go.jp (K.S.); nagafune.ikuko@qst.go.jp (I.N.); wandalila@nm.gov.my (W.D.W.C.); 2 Institute of Agriculture, Tokyo University of Agriculture and Technology, 3-5-8 Saiwai-cho, Fuchu 183-8509, Tokyo, Japan; takeda.kiyoko1228@gmail.com (K.T.); nohtsu@cc.tuat.ac.jp (N.O.-O.); sokazaki@cc.tuat.ac.jp (S.O.); tadashiy4795@gmail.com (T.Y.); 3 Agrotechnology & Bioscience, Malaysian Nuclear Agency, Kajang 43000, Selangor, Malaysia

**Keywords:** *Bradyrhizobium diazoefficiens*, high-temperature tolerance, ion beam, mutant, inversion, whole-genome sequencing, RNA sequencing

## Abstract

Biofertilizers are promising technologies for achieving sustainable agriculture. However, high-temperature tolerance is a constraint that limits the function of microbial inoculants. To characterize the genetic changes responsible for the high-temperature tolerance of rhizobia, mutant screening was performed using *Bradyrhizobium diazoefficiens* USDA110. The wild-type cells were mutagenized with carbon-ion irradiation, and two mutant strains, designated M10 and M14, were obtained after a three-day heat-shock treatment at 43 °C. In particular, M14 showed superior growth at 36 °C, at which temperature growth of the wild type was extremely slow, whereas M14 grew more slowly than the wild type at 32 °C. Whole-genome sequencing revealed that M10 had seven point mutations, whereas M14 had eight point mutations together with a 1.27 Mb inversion. RNA sequencing showed that the number of differentially expressed genes greatly exceeded the actual number of induced mutations. In M14, a gene cluster associated with pyruvate metabolism was markedly downregulated, probably because of disjunction with the promoter region after inversion, and was considered to be the cause of the slow growth rate of M14 at 32 °C. Notably, transmembrane proteins, including porins, were enriched among the genes upregulated in both M10 and M14. M14 was confirmed to retain symbiotic functions with soybeans. These results indicate that high-temperature tolerance was conferred by random mutagenesis while the symbiotic functions of rhizobia was maintained.

## 1. Introduction

Agricultural food production has been greatly increased through the use of pesticides and chemical fertilizers. However, their overapplication has serious environmental impacts [1]. Application of biofertilizers, in contrast, is a preferential strategy that provides many direct and indirect benefits for mitigating the adverse effects of chemical fertilizers [2]. Biofertilizers are materials that contain living microorganisms, such as arbuscular mycorrhizal fungi, plant growth-promoting rhizobacteria, and nitrogen-fixing rhizobia, which have the ability to enhance plant nutrient uptake and availability by colonizing the rhizosphere or root tissues [3,4]. In particular, rhizobia elicit the formation of specialized organs termed nodules on the roots of their leguminous hosts and catalyze the reduction of atmospheric nitrogen to ammonia [5]. Interestingly, biofertilizers increase crop yields not only by improving nutrient availability but also by increasing the production of phytohormones and enhancing stress tolerance, and their continued use is expected to improve soil health [6]. Moreover, the use of effective rhizobia as biofertilizer inoculants with leguminous plants, an economically important crop, could reduce not only the chemical fertilizers but also the energy inputs and greenhouse gas emissions associated with their production and application [7]. The utilization of biofertilizers has attracted attention because of the heightened interest in environmentally sustainable development, but their widespread use is hampered by several problems. High temperature is one of the major constraints and limits the functions of microbial inoculants in relation to their growth, viability, and nitrogen-fixing ability after biofertilizer application, together with their viability during storage and transportation [8,9,10,11]. Therefore, the development of high-temperature-tolerant biofertilizer inoculants is important for improving crop productivity under climate change.

Regarding the high-temperature tolerance of rhizobia, 42 strains of *Rhizobium japonicum* have been assessed, and the maximum permissive temperature was reported to be in the range of 29.8–38.0 °C [12]. In addition, two strains of *Rhizobium leguminosarum* bv. *viceae* have been reported to be heat tolerant up to 42 °C [13]. Considerable research effort has been devoted to isolating high-temperature-tolerant strains from nodules collected in different areas [14,15,16]. These preceding studies indicate that the high-temperature tolerance varies among species and strains, and some regional characteristics have been suggested. In addition, a correlation between the tolerance and the degree of induction of chaperone genes in response to heat stress has been suggested [15]. Mutant screening is a useful approach to obtain novel genetic resources and to understand the underlying mechanisms of a phenotype of interest. However, screening for high-temperature-tolerant mutants of rhizobia has been conducted in only a small number of studies [9,17]. Based on the assumption that changes in DNA gyrase and the resulting DNA conformation are important factors in high-temperature tolerance, high-temperature-tolerant mutants of *R*. *leguminosarum* bv. *phaseoli* were obtained after nitrous acid mutagenesis by using a selection medium containing a DNA gyrase inhibitor, although a detailed analysis has not been performed [9]. High-temperature-tolerant strains of *Rhizobium* sp. (*Cajanus*) were obtained after heat shock at 43 °C for 6 h, and some showed a favorable symbiotic performance under heat stress [17].

Ion beams are a more powerful mutagen, compared with gamma rays or X-rays, as they produce dense and localized ionization along the path of the ion particles. Ion beams have been applied for mutant screening in higher plants and microorganisms [18,19,20]. Although the biological effect of ion beams varies depending on the energy and nuclide, the value of linear energy transfer (LET), which indicates the amount of energy transferred to the irradiated material per unit length, is the most crucial measure of their biological effect. Recently, we performed the first systematic study of ion-beam-induced mutation in bacteria using *Bacillus subtilis* as a model sample [21]. It was shown that the ion beams tended to induce drastic changes, such as large deletions or inversions, more frequently as the LET value increased; however, an extremely high LET value was ineffective because the lethality was excessive. It was concluded that ion beams with a LET value of approximately 111 keV/µm induced the most diverse mutation types and would be useful for mutagenesis.

In this study, high-temperature-tolerant mutants of *Bradyrhizobium diazoefficiens* strain USDA110 were obtained by ion-beam irradiation. *Bradyrhizobium diazoefficiens* USDA110 is a soybean-nodulating rhizobium that has been widely used in the fields of molecular genetics, physiology, and ecology owing to its superior symbiotic function and the availability of a single circular genomic sequence [22,23]. The obtained mutants were characterized by their biological characteristics, DNA mutation sites, and changes in the transcriptome to elucidate the genetic changes responsible for high-temperature tolerance.

## 2. Materials and Methods

### 2.1. Mutagenesis

A laboratory strain of *Bradyrhizobium diazoefficiens* strain USDA110, which was originally provided by the United States Department of Agriculture, was used. Cells were grown in yeast–mannitol (YM) broth (0.05% K_2_HPO_4_, 0.02% MgSO_4_, 0.01% NaCl, 1.0% mannitol, and 0.05% yeast extract) with agitation at 30 °C for 5 days to the stationary phase (~10^8^ colony forming units [CFU]/mL). Cells were collected by centrifugation at 5000× *g* for 7 min at 4 °C, rinsed with sterilized water, and resuspended in a solution containing 1.0% skim milk and 1.5% glutamic sodium with a final cell density of ~10^9^ CFU/mL. The suspension (100 µL) was dropped onto a sterilized cellulose membrane filter (25 mm diameter, pore size 0.22 µm; EMD Millipore Corp., Billerica, MA, USA) and placed in a sterilized polypropylene case (30 mm × 30 mm). The membranes were frozen at −80 °C for 60 min and dried for 90 min using a freeze dryer (FDU-2200, EYLA, Tokyo, Japan). The freeze-dried membranes were covered with 7.5-µm-thick Kapton polyimide film (Toray-Dupont, Tokyo, Japan) and irradiated with 0–500 Gy of carbon ions (LET 156 keV/µm) at the Takasaki Institute for Advanced Quantum Science, National Institutes for Quantum Science and Technology (Takasaki, Gunma, Japan) at room temperature. In total, 462 membranes were irradiated in eight independent experiments. The irradiated cells on a membrane filter were transferred to 2.0 mL microtubes and resuspended in 1.0 mL YM broth by vigorous shaking. Then, 0.9 mL cell suspension was mixed with 2.1 mL YM broth in a 15 mL test tube and grown with agitation at 30 °C for 5–7 days, depending on the irradiation dose to the stationary phase. The mutagenized cells were stored at −80 °C after addition of glycerol to a final concentration of 14%.

### 2.2. Screening of High-Temperature-Tolerant Mutants

The mutagenized cells were grown in 3 mL YM broth in a 15 mL test tube at 30 °C for 5 days until the stationary phase with agitation. The culture solution was incubated at 43 °C for 3 days. The cells were collected by centrifugation at 5000× *g* for 7 min at 4 °C, spread on YM plates (YM broth solidified with 1.5% bacto agar), and incubated at 30 °C for 7 days. The individual colonies were picked up and grown in 2 mL YM broth at 30 °C and stored as glycerol stock at −80 °C.

### 2.3. Evaluation of High-Temperature Tolerance on Agar Plates

The wild-type and mutant cells were precultured in modified peptone–salt–yeast extract (mPSY) broth (0.3% peptone, 0.01% NaCl, 0.1% yeast extract, 0.01% MgSO_4_·7H_2_O in 3 mM potassium phosphate buffer [PB], pH 6.8) with agitation at 32 °C for 3 days. A serial dilution was prepared with PB using cells in the exponential phase and spotted on mPSY plates solidified with 1.5% bacto agar. Congo red was added to the mPSY plate at a concentration of 0.0025% to visualize colonies clearly. The agar plates were incubated at 29, 32, 34, or 36 °C for up to 9 days.

### 2.4. pH Change of YM Medium

To check the pH change of the growth medium, the precultured cells of the wild-type and mutant strains were resuspended in 2 mL YM broth containing 0.15% bromothymol blue with a cell density of approximately 10^6^ CFU/mL and incubated with agitation at 32 °C for 4 days. This experiment was also performed using YM broth containing 1.0% glucose or 0.3% peptone instead of mannitol as a carbon source.

### 2.5. Generation Time

To evaluate the generation time, precultured cells of the wild-type, M10, and M14 strains were inoculated in 50 mL mPSY broth in a 200 mL flask with a cell density of approximately 10^4^ CFU/mL in three replicates. The cells were grown with agitation at 32 °C, and the CFU were determined twice daily for 5 days by the dilution plate method as described elsewhere [24]. The slope (*m*) of the growth curve in the exponential phase was estimated by linear regression, and the generation time was calculated as log(2)/*m*.

### 2.6. Whole-Genome Sequencing

Total genomic DNA was extracted from cultured cells using the Wizard Genomic DNA Purification Kit (Promega K.K., Tokyo, Japan). Sequencing libraries were prepared using the KAPA HyperPlus kit, KAPA universal adapter, and UDI primer mixes (Nippon Genetics Co., Ltd., Tokyo, Japan). Sequencing was performed by Novogene (Beijing, China) using a NovaSeq 6000 system, and 150 bp paired-end reads were generated. Low-quality reads were removed using Illumiprocessor (version 2.0.9). The clean reads were mapped to the USDA110 reference genome sequence (9,105,828 bp) [22] using BWA (version 0.7.5), SAMtools (version 1.3.1), and Picard-tools (version 1.119). The mean depth of coverage was in the range of 69 to 145. To detect the various types of mutations, candidate mutation sites were detected using five algorithms: GATK Haplotype Caller (version 4.3), Pindel (version 0.2.4), BreakDancer (version 1.4.5), Manta (version 1.6.0), and Lumpy (version 0.3.1), as previously described [25]. The most recent gene annotation information was obtained from the RefSeq database (accession GCF_000011365.1; https://www.ncbi.nlm.nih.gov/refseq/ accessed on 30 August 2024). For mutation detection using GATK, the candidate mutation sites common to all strains were excluded as background mutations, and then the candidate mutation sites with allele frequencies higher than 80% were confirmed with Integrative Genomics Viewer (IGV; version 2.15.4). For mutation detection using Pindel, BreakDancer, Manta, and Lumpy, the candidate mutation sites unique to a single sample were selected and confirmed by IGV.

### 2.7. RNA Sequencing

The precultured wild-type, M10, and M14 cells were spread on mPSY plates with a cell density of ~200 colonies per 9 cm dish and grown at 32 or 35 °C for 4 days. Cells were collected after gently mixing with 2 mL PB using a spreader. Total RNA was isolated using the RNeasy Protect Bacteria Mini Kit (QIAGEN K.K., Tokyo, Japan). DNA was eliminated using the DNA-free DNA Removal Kit (Thermo Fisher Scientific K.K., Tokyo, Japan). RNA sequencing was conducted with two replicates for cells cultured at 32 °C and a single sample of cells cultured at 35 °C. The sequencing library was prepared by Genome-Lead Co., Ltd. (Takamatsu, Kagawa, Japan) using the NEBNext rRNA Depletion Kit (Bacteria) (New England Biolabs Japan, Inc., Tokyo, Japan) and the MGIEasy RNA Directional Library Prep Set (MGI Tech Co., Ltd., Shenzhen, China). The 150 bp paired-end reads of 5.3–5.7 Gb per sample were generated using DNBSEQ-T7RS (MGI Tech). Quality control and trimming were performed using fastp (version 0.23.2). The clean reads were mapped to the USDA110 reference genome sequence using HISAT2 (version 2.2.1). The number of reads mapped to each gene was counted using featureCounts (version 2.0.1), and the count data were normalized using edgeR (version 3.42.4), which employs the trimmed mean of the M-values. The count data for 21 genes out of the total data for 8577 genes (8331 genes and 246 pseudogenes) were removed because the count was zero in at least one of the nine samples. The count data were analyzed using iDEP2.01 [26].

### 2.8. Symbiotic Function

Soybean (*Glycine max* ‘Enrei’) seeds were surface sterilized with 0.5% sodium hypochlorite solution for 5 min and rinsed with pure water several times. The seeds were germinated on moistened tissue paper on a tray at 25 °C for 3 days in darkness. The germinated seedlings were transferred to a growth pouch (Daiki Rika Kogyo Co., Ltd., Saitama, Japan), inoculated with 2 × 10^9^ wild-type or M14 cells per seedling, and grown using nitrogen-free nutrient solution at 25 °C in a growth chamber for 4 weeks under a 16 h/8 h (light/dark) photoperiod [27]. The whole plants were harvested and washed in running tap water. The number of nodules and the fresh and dry weights of plant shoots, roots, and root nodules were measured. To evaluate nitrogenase activity, the acetylene reduction activity was determined as described elsewhere [28,29].

## 3. Results

**Figure 1:**
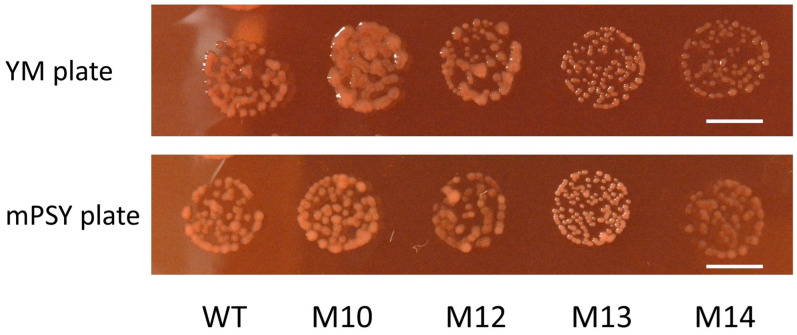
Colonies of the wild-type and mutant strains grown on YM and mPSY agar plates at 32 °C for 5 days. Bars = 5 mm.

To obtain high-temperature-tolerant mutants, the wild-type USDA110 was mutagenized with carbon ion beams. Survival was reduced to approximately 10% and 1% after irradiation with 200 Gy and 400 Gy, respectively (Figure S1). As a first screening, the culture solution of mutagenized cells was incubated at 43 °C for 3 days, and then the cells were spread on agar plates and incubated at 30 °C for 7 days. Thirty-seven colonies were initially obtained as mutant candidates. Of this number, 16 colonies, in which the 16S ribosomal DNA sequence was identical to that of the wild type, were further examined. We used YM broth for mutant screening; however, the relationship between OD_600_ and CFU was often unstable. We speculated that this was partly due to the difference in the released amount of extracellular polysaccharides (EPSs). Therefore, we used mPSY broth in the following experiments unless otherwise indicated. The released amount of EPSs greatly differed among the mutant strains on YM plates, but this difference was diminished on mPSY plates (Figure 1).

**Figure 2:**
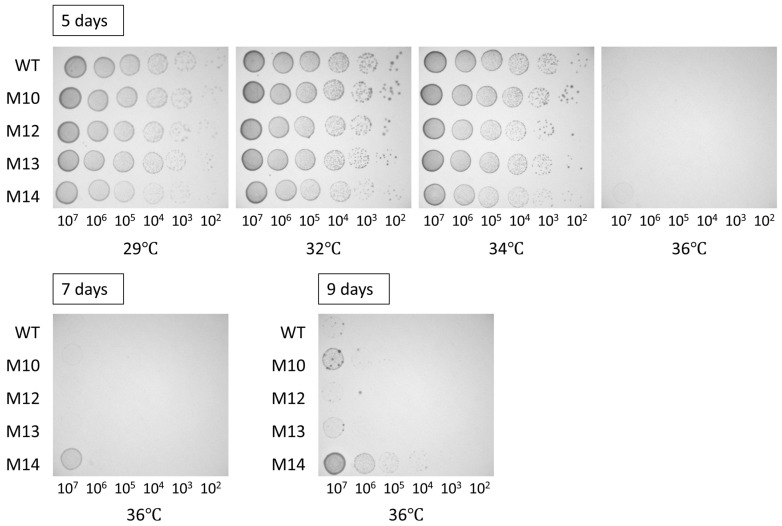
Evaluation of high-temperature tolerance in a serial dilution spotting assay. Ten microliters of culture solution of the wild-type and mutant strains in the range of 10^2^–10^7^ CFU/mL were spotted on mPSY agar plates and incubated under the indicated temperature for up to 9 days.

To confirm the high-temperature tolerance, a serial dilution was prepared using the cells of the wild-type and mutant candidates and spotted on mPSY plates. After 5 days at 32, 34, or 36 °C, two mutant strains, designated M10 and M14, were indicated to have a greater high-temperature tolerance than the wild type (Figure 2). The M10 and M14 strains were derived from the same sample irradiated with 300 Gy. Two additional mutant strains, M12 and M13, were also derived from the identical sample; therefore, some of the four strains might represent the same mutant. Therefore, these four mutant strains were confirmed again for the high-temperature tolerance. As shown in Figure 2, M10 and M14, particularly the latter, clearly showed superior growth compared with the wild type at 36 °C. Thus, the characteristics of M10 and M14 were further examined. The optimal growth temperature was ~32 °C for all strains.

**Figure 3:**
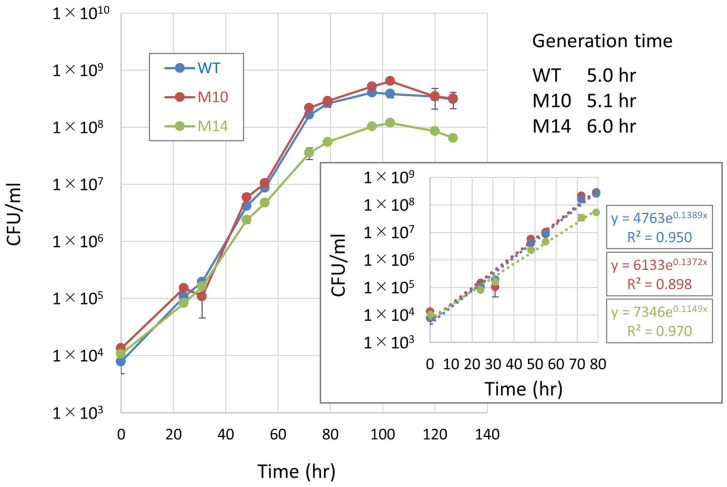
Growth curve of the wild-type, M10 and M14 strains. Generation time was estimated from the slope in the exponential phase.

**Figure 4:**
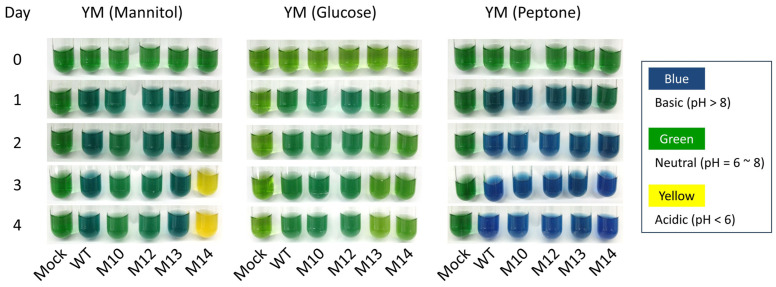
Change in pH of the growth medium during cell growth. The wild-type and mutant strains were grown in YM broth containing bromothymol blue at 32 °C for 4 days. The original YM broth containing mannitol, as well as YM broth containing glucose or peptone instead of mannitol, were used.

In addition to the difference in high-temperature tolerance, M10 released a greater amount of EPSs, whereas M13 released much fewer compared with the wild type (Figure 1). M14 formed a smaller colony at 32 °C, suggesting that the growth rate was lower than that of the other strains (Figure 2). Consistent with this observation, the estimated generation times of the wild type and M10 were 5.0 and 5.1 h, respectively, whereas that of M14 was 6.0 h (Figure 3). *Bradyrhizobium* strains, including USDA110, alkalize the YM broth [30]; however, M14 acidified the YM broth (Figure 4). These observations suggested that the four mutant strains were independent strains with distinct biological characteristics. This acidification was considered to be mannitol-dependent because M14 showed a similar trend of pH change to that of the other strains when the carbon source was changed to glucose or peptone.

**Figure 5:**
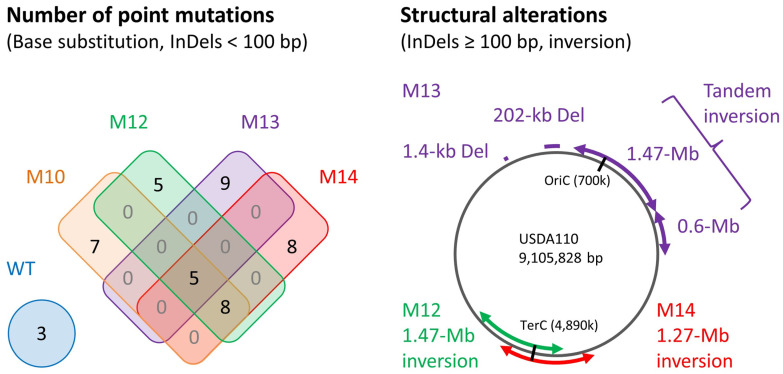
Venn diagram of the number of point mutations (single-base substitutions [SBSs] and insertions/deletions [Indels] < 100 bp) and schematic representation of structural alterations detected by whole-genome sequencing analysis. No structural alteration was detected in M10.

Whole-genome sequencing (WGS) analysis was performed to determine the mutations induced by carbon ions. The number of point mutations, i.e., single-base substitutions (SBSs) and insertions/deletions (Indels) < 100 bp, that were specific to each mutant strain was in the range of 5 to 9 (Figure 5). Five mutations were common to the four mutant strains, and eight mutations were common to three mutant strains except M10. Three mutations were specific to the wild type. The SBSs comprised 76% of the total point mutations, and the remainder were Indels of 1–15 bp (Figure S2). Structural alterations were detected in three mutant strains except M10 (Figure 5). A 1.27 Mb inversion was observed in M14, and a 1.47 Mb inversion was detected in M12. A 202 kb deletion, a 1.4 kb deletion, as well as a tandem inversion composed of 1.47 Mb and 0.6 Mb regions were detected in M13. The loss of 186 genes was caused by the 202 kb deletion in M13. The TCAAA sequence motif was observed in the two rejoined sites of the 1.27 Mb inversion of M14 (Figure S3). The microhomology in the rejoined site was also observed in the 202 kb and 1.4 kb deletions of M13, suggesting that those sequences were employed in the rejoining of the broken DNA ends. The induced mutations were indicated to affect the amino acid sequence of six and four genes in M10 and M14, respectively (Table 1). All mutations detected by the WGS analysis and the deduced effects on annotated genes are listed in Table S1.

**Figure 6:**
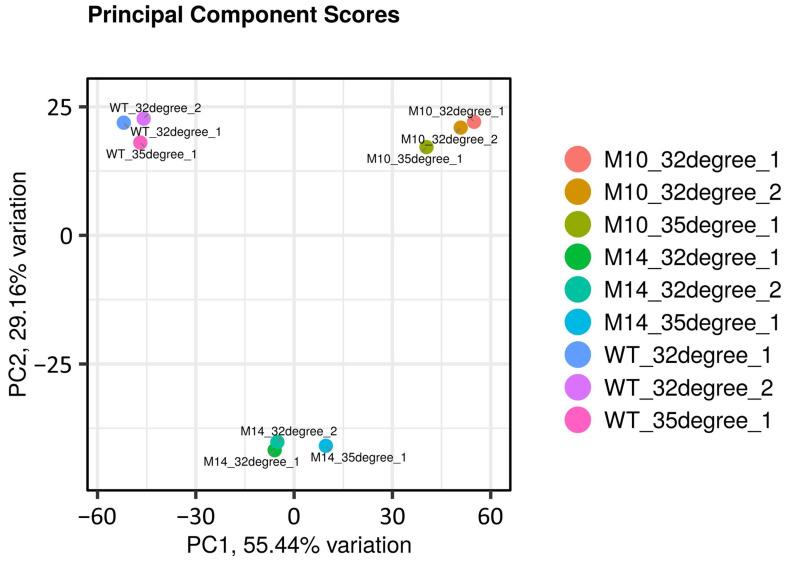
Principal component analysis of the RNA-sequencing data.

**Figure 7:**
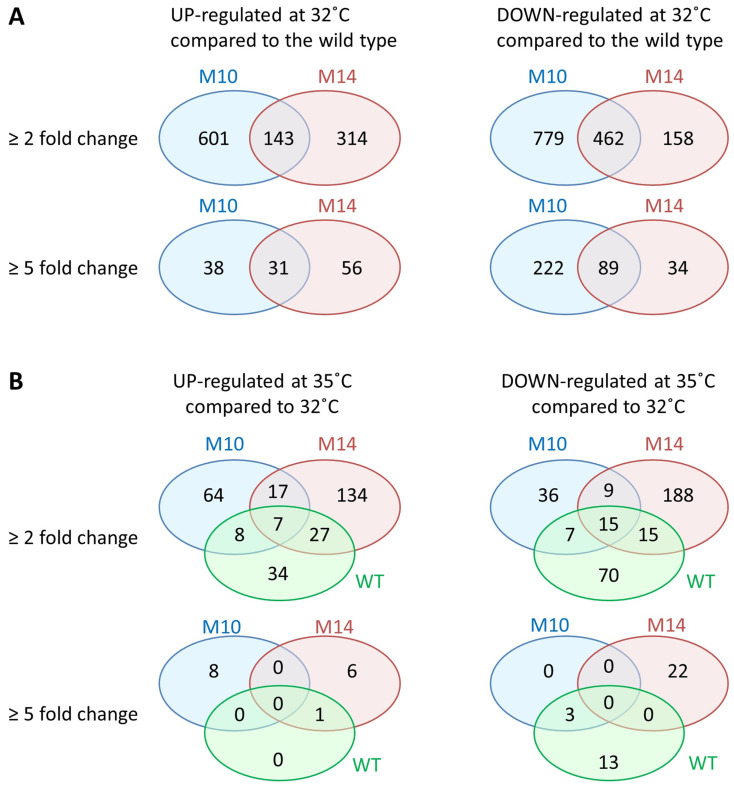
Number of differentially expressed genes (DEGs). (**A**) Number of DEGs in the M10 and M14 strains compared with the wild type at 32 °C. (**B**) Number of DEGs at 35 °C compared with 32 °C for each strain.

RNA-sequencing (RNA-seq) analysis was performed to examine the effects on the gene expression profile using the wild-type, M10, and M14 cells grown on mPSY plates at 32 or 35 °C. Principal component analysis (PCA) of the expression profile indicated that the difference in gene expression between the strains was much greater than the difference between the 32 and 35 °C treatments (Figure 6). The M10 strain was characterized by a greater number of downregulated genes compared with that of M14 at 32 °C (Figure 7A). As suggested by the PCA, the number of differentially expressed genes (DEGs) between 32 and 35 °C was much smaller than the number of DEGs between strains (Figure 7B). Nevertheless, RNA-seq analysis revealed that the expression of a large number of genes was affected, although the total number of induced mutations was only 20 or less in the M10 and M14 genomes.

**Figure 8:**
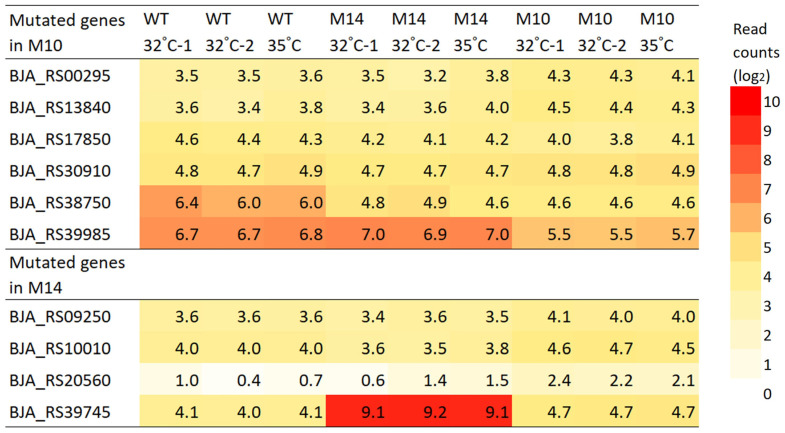
Expression level of mutated genes in the M10 and M14 strains. The values are the log_2_ value of the read counts.

**Figure 9:**
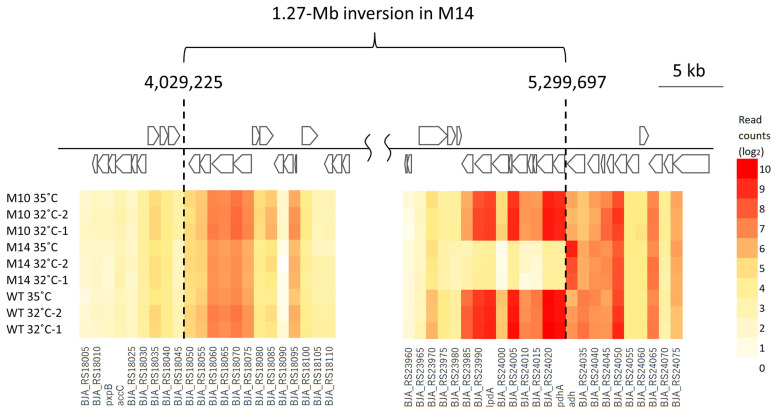
Schematic representation of gene arrangement and expression levels in the vicinity of the breakpoint of the 1.27 Mb inversion in M14. Numbers indicate the position in the USDA110 reference genome sequence. The arrows indicate the coding sequence and the direction of transcription. The color scale indicates the log_2_ value of the read counts.

**Figure 10:**
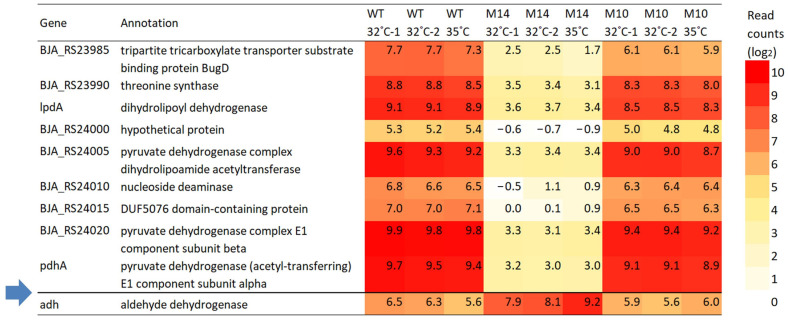
Expression level of genes in the vicinity of the breakpoint of the inversion at position 5,299,697 in M14. The blue arrow indicates the position of the breakpoint. The values are the log_2_ value of the read counts.

Figure 8 shows the expression level of mutated genes in M10 and M14. The *BJA_RS39745* (old locus tag: bll7831) gene was upregulated in M14 by approximately 34 times (shown as log_2_-transformed values in Figure 8). The expression levels of the other mutated genes were more or less similar to those in the wild type. The two breakpoints of the 1.27 Mb inversion in M14 were not within the coding sequence; however, the expression level of nine genes in the vicinity of the breakpoint at position 5,299,697 was strongly affected (Figure 9). This breakpoint was located 44 bp upstream of *pdhA* and 183 bp downstream of *adh*. Figure 10 shows the expression level of the genes on either side of this breakpoint. The four genes *pdhA* (bll4783), *BJA_RS24020* (bll4782), *BJA_RS24005* (bll4779), and *lpdA* (bll0449) are considered to be involved in the process of pyruvic acid decarboxylation resulting in the synthesis of acetyl-CoA, which is needed at the first step of the tricarboxylic acid cycle. The downregulation of these genes might be associated with the slow growth of M14 at 32 °C.

To confirm whether the M14 strain retains the symbiotic function, an inoculation test with soybean was performed. The M14-inoculated plants showed a higher number of nodules per plant than the wild type (Table 2). No significant difference was observed in the fresh weight of nodules, shoots, and roots or in acetylene reduction activity. These results suggested that the M14 strain retained the symbiotic function.

## 4. Discussion

In this study, high-temperature-tolerant mutants of *B. diazoefficiens* USDA110 were obtained by carbon-ion irradiation. The WGS and RNA-seq analyses were performed to detect genetic changes responsible for the tolerance. Irradiation doses resulting in a survival rate of 1% to 10% have been empirically considered to be the most effective range of doses for mutant screening of bacteria [20]. The M10 and M14 mutants were obtained from the sample irradiated with 300 Gy, which resulted in a survival rate of approximately 4%. Unexpectedly, the four mutant strains had five mutations in common that were absent in the wild type. In addition, eight point mutations were common to three mutant strains. It is plausible that these mutations existed in the original wild-type cells used for mutagenesis because induction of the same set of mutations in independent cells is improbable. More than 60 variations were common to both the wild-type and the four mutant strains in comparison with the reference genome sequence (Table S2). This fact strongly supports the derivation of the wild-type and four mutant strains from the same laboratory strain. It was assumed that three specific mutations detected in the wild type were spontaneous mutations that occurred during subculture.

The four mutant strains harbored 5–9 point mutations (Figure 5). The frequency of these mutations (0.6–1.0 × 10^−6^/bp) is comparable to the mutation frequency (0.5–1.2 × 10^−6^/bp) observed in *Bacillus subtilis* after carbon-ion irradiation in a previous study [21]. The SBSs comprised 76% of the total point mutations (Figure S2), which was similar to the types of mutations observed in *B. subtilis* [21]. Interestingly, three of the four mutant strains had structural alterations (Figure 5). We usually irradiate freeze-dried bacterial cells to maximize the dense ionization of carbon ions [20] because water enhances an indirect radiation action by generating highly reactive free radicals [31,32]. This suggests that carbon-ion irradiation of freeze-dried cells is prone to induce structural variation. Structural variations have also been observed after carbon-ion irradiation of freeze-dried cells of *Aspergillus oryzae* and *B. subtilis*, although the frequency was relatively low (2–3 of 13 lines) [21,33]. The structural alterations originate from the mis-rejoining of broken DNA ends. Three of the five structural alterations observed in this study had a microhomology of 3–13 bp in the rejoined site (Figure S3). This suggests the involvement of the non-homologous end joining (NHEJ) pathway, which directly ligates DNA ends relying on short or no homology [34]. The Ku complex, ligase, and other components involved in the NHEJ pathway are conserved in many bacterial species, including rhizobia [35,36].

The M10 cells release a very high amount of EPSs, whereas M13 cells release few EPSs, compared with that of the wild type (Figure 1). The EPSs of *Rhizobium* are suggested to have multiple functions, such as in symbiosis with the host plant, biofilm formation, and protection against environmental stresses [37]. The EPSs of USDA110 are reportedly composed of mannose, 4-*O*-methylgalactose (or galactose), glucose, and galacturonic acid [38,39]. Therefore, the cells release higher amounts of EPSs when they are grown with YM broth containing mannitol, compared with mPSY broth containing peptone as a carbon source. The USDA110 genome has a putative gene cluster composed of 21 genes (*BJA_RS11550* [blr2358] to *BJA_RS11650* [bll2378]) involved in the EPS synthesis pathway [22,40]. The expression level of 11 of the 21 genes was two- to five-fold higher in both M10 and M14 compared with that of the wild type (Table S3). It is notable that the expression level of seven genes in a separate putative gene cluster associated with mannitol metabolism (*BJA_RS34610* [bll6830] to *BJA_RS34640* [blr6836]) was eight- to 16-fold higher in M10 than in the wild type, whereas the expression level in M14 was comparable to that in the wild type (Table S3). The high production of EPSs is suggested to be the cause of the heat-tolerant mutant of *Rhizobium* sp. (*Cajanus*) [41]. The high expression levels of genes involved in mannitol metabolism may be associated with high EPS production and the high-temperature tolerance of M10. In contrast, M13 cells produce few EPSs, even if grown in YM broth (Figure 1). Only M13 had experienced a significant loss of genetic information, comprising 186 genes in a 202 Mb deletion, among the four mutant strains, which might reflect the limited EPS production of M13 cells.

Although the exact reason for the high-temperature tolerance of M10 and M14 remains unclear, M14 is suggested to have a different tolerance mechanism other than EPSs because M14 shows higher heat tolerance but produces much fewer EPSs compared with M10 under the present experimental conditions. It is reasonable to consider that the mutated genes are the primary cause of the high-temperature tolerance of M10 and M14. The number of strain-specific mutations in the coding sequence of annotated genes was six in M10 and four in M14 (Table 1). Of these, only *BJA_RS39745* [bll7831], which encodes a MarR family transcriptional regulator, showed a marked upregulation by approximately 34 times (Figure 8, Table S3). Interestingly, an additional two immediately downstream genes (*BJA_RS39750* [blr7832] and *BJA_RS39755* [blr7833]), which are suggested to have ABC-type transporter activity, were also upregulated by approximately 30 times (Table S3). These changes might be associated with the high-temperature tolerance of M14, although the specific function of these genes cannot be easily deduced because MarR family transcriptional regulators comprise a superfamily of 36 genes, and a total of 279 genes are associated with ABC-type transporter activity in the USDA110 genome.

The present RNA-seq analysis showed that a large number of genes were differentially expressed in M10 and M14 compared with the number of actually mutated genes (Figure 7). In addition, the difference in DEGs between strains was much greater than that between 32 and 35 °C. Thirty-one genes were upregulated in both M10 and M14 by five-fold or more at 32 °C (Figure 7A). It is notable that eight of these 31 genes encode a transmembrane protein, including porins. These changes are worth further investigation because the significant production of transmembrane proteins, such as porin, reportedly confers high-temperature tolerance in *Salmonella typhimurium* and *Escherichia coli* [42,43]. Furthermore, the RNA-seq analysis revealed the downregulation of nine genes located in the vicinity of one of the two breakpoints of the 1.27 Mb inversion in M14 (Figure 9 and Figure 10). These genes are tightly located in the same orientation, suggesting that they are controlled by a common regulator. The most probable promoter sequence, deduced by the similarity to the promoter sequences of *E. coli* [44], is the −35 (CTGATC) and −10 (AATATT) regions separated by a 17 bp spacer at nucleotide positions 5,299,768 to 5,299,796, which is located immediately upstream of these genes but was disconnected as a result of the inversion. According to the current gene annotations, at least four of the nine genes are involved in pyruvate metabolism, which contributes to the energy production system. An *E. coli* strain carrying a mutated pyruvate dehydrogenase reportedly accumulates pyruvate and shows a lower growth rate than the wild type [45]. Reduced energy production due to the downregulation of pyruvate metabolism is most likely the cause of the slow growth of M14 at 32 °C. The relationship with high-temperature tolerance will be examined by a complementation test.

The correlation between heat tolerance and the degree of induction of chaperone genes has been suggested in rhizobia [15]. The USDA110 genome has twelve putative chaperone genes including seven *groEL* (*BJA_RS10080* [bll2059], *BJA_RS18230* [blr3683], *BJA_RS23230* [blr4635], *BJA_RS26360* [blr5227], *BJA_RS28445* [bsl5624], *BJA_RS35340* [blr6979] and *BJA_RS38205* [blr7533]), four *dnaJ* (*BJA_RS00915* [bll0184], *BJA_RS03435* [bll0680], *BJA_RS25995* [bll5157] and *BJA_RS33790* [bll6670]) and one *dnaK* (BJA_RS03420 [bll0677]). Of those, only *groEL* (*BJA_RS18230* [blr3683]) showed more than 5-fold downregulation in both M10 and M14, and the expression levels of the others were comparable to that of the wild type (Table S3). Thus, no positive relationship was found between the chaperone genes and high-temperature tolerance in this study.

It was confirmed that the M14 strain retained the symbiotic functions with soybean. M14 is not suitable for practical use owing to its low growth rate. However, the present results strongly suggest that high-temperature tolerance can be conferred by mutagenesis while maintaining the symbiotic functions. The high-temperature tolerance of M10 and M14 is stable so far after several rounds of subculturing from a frozen stock. However, the stability should be further confirmed because the acquired properties may be lost after a long time. At present, the exact reason for the high-temperature tolerance of M10 and M14 remains unclear. The fact that the number of DEGs greatly exceeded the actual number of induced mutations also makes it difficult to determine the genetic changes responsible for the high-temperature tolerance. Nevertheless, further mutant isolation and characterization of the phenotype, including field inoculation trials, would provide important insights for verifying its effectiveness in mitigating reduced crop productivity under climate change [46].

## Figures and Tables

**Table 1:** Deduced amino acid alterations of annotated genes in the M10 and M14 strains.

M10 Strain				
Position	Mutation	Gene	Annotation	Alteration
53,830	GC > CG	BJA_RS00295	DUF2493 domain-containing protein	Asp36His
3,068,840	GC > CG	BJA_RS13840	MBL fold metallo-hydrolase	Ala59Gly
3,985,396	AT > GC	BJA_RS17850	Serine/threonine-protein kinase	Asn192Asp
6,724,936	AT > CG	BJA_RS30910	SOS response-associated peptidase	Asn128Thr
8,383,126	AT > CG	BJA_RS38750	PQQ-binding-like beta-propeller repeat protein	Leu71Arg
8,637,494	−1 bp	BJA_RS39985	Adenylate/guanylate cyclase domain-containing protein	frameshift
**M14 strain**				
**Position**	**Mutation**	**Gene**	**Annotation**	**Alteration**
2,002,453	AT > GC	BJA_RS09250	Hypothetical protein	His177Arg
2,201,526	GC > AT	BJA_RS10010	GFA family protein	Ala332Thr
4,577,785	AT > CG	BJA_RS20560	Sugar ABC transporter permease	Met63Leu
8,585,966	GC > AT	BJA_RS39745	MarR family transcriptional regulator	Thr132Ile

**Table 2:** Symbiotic function of the M14 strain in inoculated soybean.

Strain	No. of Nodules per Plant	Nodule Fresh Weight		Shoot Dry Weight (mg)	Root Dry Weight (mg)	Acetylene Reduction Activity (mol/h/g Nodule Fresh Weight)
		(mg/Plant)	(mg/Nodule)			
WT	32.0 ± 9.9 ^a^	173 ± 46 ^a^	5.5 ± 0.8 ^a^	482 ± 110 ^a^	195 ± 47 ^a^	13.6 ± 7.8 ^a^
M14	52.0 ± 7.9 ^b^	177 ± 42 ^a^	3.5 ± 1.1 ^a^	545 ± 78 ^a^	232 ± 43 ^a^	6.5 ± 4.8 ^a^

Values are the mean ± standard deviation (*n* = 6). Different letters within a column indicates a significant difference (Student’s *t*-test, *p* < 0.05).

## Data Availability

The raw sequencing data were deposited in the DNA Data Bank of Japan Sequence Read Archive (https://ddbj.nig.ac.jp/dra accessed on 30 August 2024) with the accession numbers PRJDB18633 and PRJDB18636.

## Supplementary Materials

The following supporting information can be downloaded at https://www.mdpi.com/article/10.3390/microorganisms12091819/s1, Figure S1: Survival curve of *B. diazoefficiens* strain USDA110 irradiated with carbon ions; Figure S2: Classification of 42 point mutations detected by WGS analysis in the M10, M12, M13, and M14 mutant strains; Figure S3: Nucleotide sequence of the rejoined site of structural alterations; Table S1: Mutations detected by WGS analysis; Table S2: Mutations detected in both the wild-type and four mutant strains by WGS; Table S3: Normalized count data for the RNA-seq analysis.
