# Supplementary figures and images for: Isolation and Characterization of High-Temperature-Tolerant Mutants of Bradyrhizobium diazoefficiens USDA110 by Carbon-Ion Beam Irradiation
Source: Microorganisms. 2024 Sep 2;12(9):1819. doi: 10.3390/microorganisms12091819 (PMC11434629; doi:10.3390/microorganisms12091819)

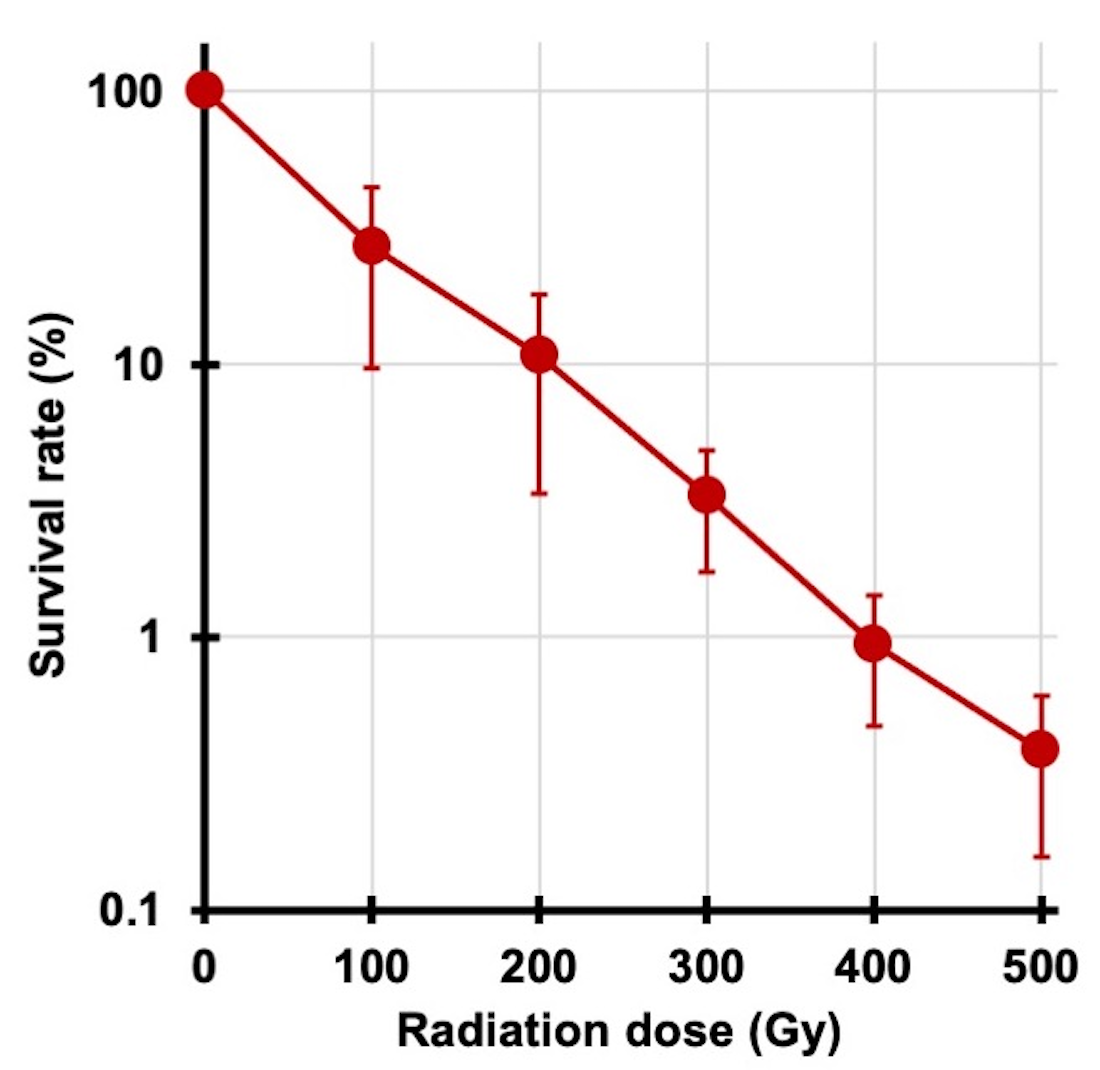

Supplement: Supplementary file 1 [file microorganisms-12-01819-s001.zip › Supplemental FigS1.tiff]

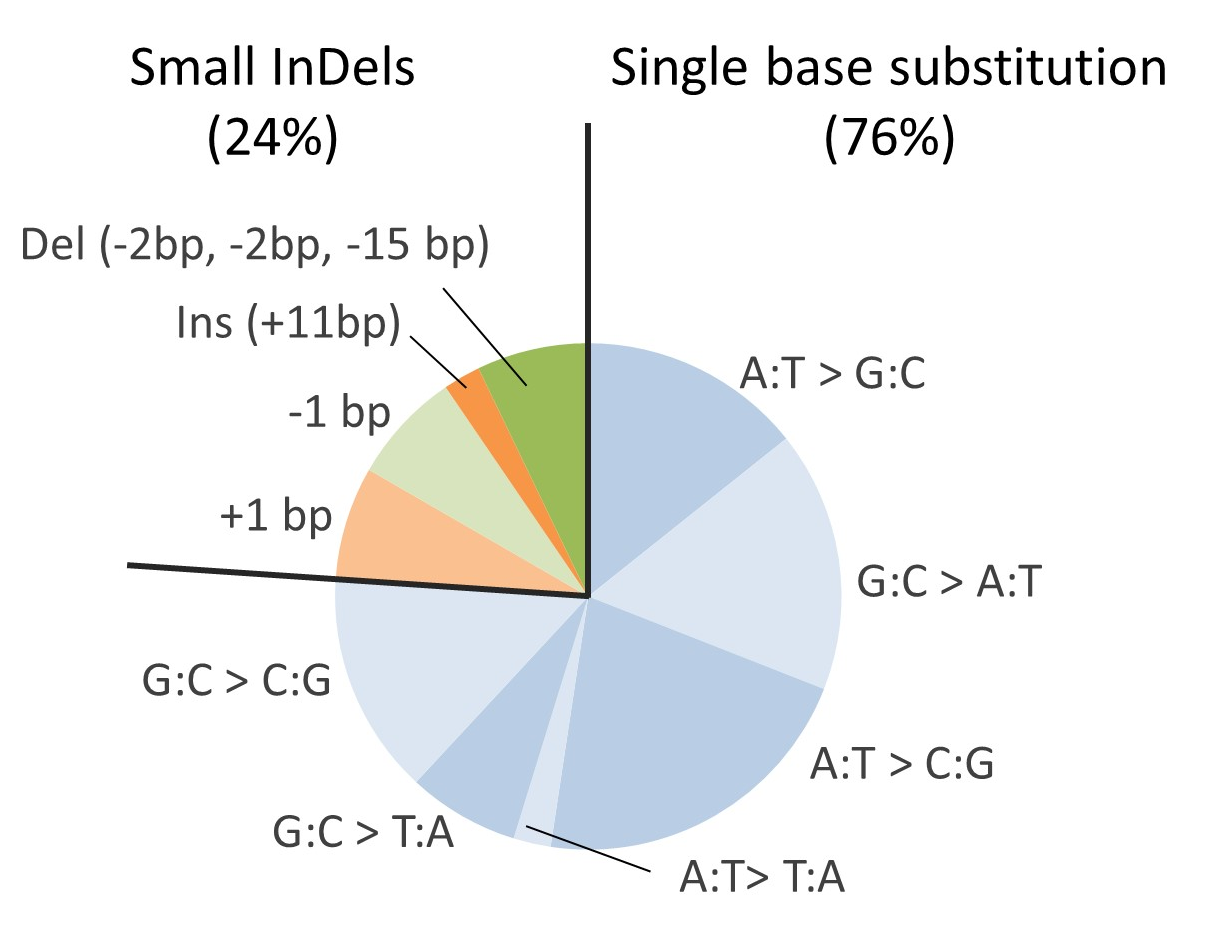

Supplement: Supplementary file 1 [file microorganisms-12-01819-s001.zip › Supplemental FigS2.tiff]

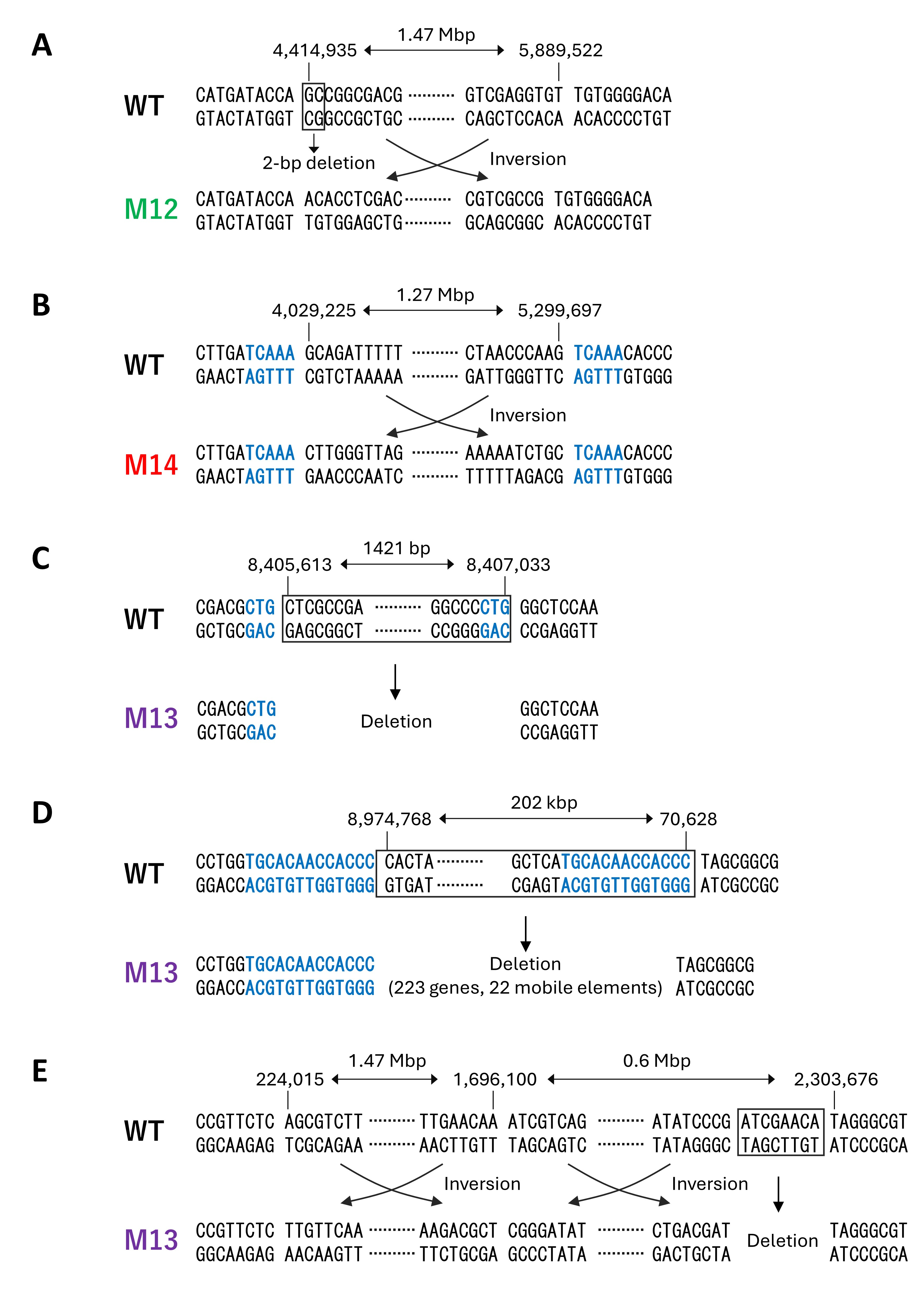

Supplement: Supplementary file 1 [file microorganisms-12-01819-s001.zip › Supplemental FigS3.tiff]
